# Review on new approach methods to gain insight into the feto-maternal interface physiology

**DOI:** 10.3389/fmed.2023.1304002

**Published:** 2023-11-30

**Authors:** Ramkumar Menon, Louis J. Muglia, Lisa Hara Levin

**Affiliations:** ^1^Department of Obstetrics and Gynecology, Division of Basic Science and Translational Research, The University of Texas Medical Branch at Galveston, Galveston, TX, United States; ^2^The Burroughs Wellcome Fund, Research Triangle Park, NC, United States; ^3^Cincinnati Children’s Hospital Medical Center, University of Cincinnati College of Medicine, Cincinnati, OH, United States; ^4^Coridea, LLC, New York, NY, United States

**Keywords:** microphysiology, organ on a chip, pregnancy, parturition, organoids

## Abstract

Non-human animals represent a large and important feature in the history of biomedical research. The validity of their use, in terms of reproducible outcomes and translational confidence to the human situation, as well as ethical concerns surrounding that use, have been and remain controversial topics. Over the last 10 years, the communities developing microphysiological systems (MPS) have produced new approach method (NAMs) such as organoids and organs-on-a-chip. These alternative methodologies have shown indications of greater reliability and translatability than animal use in some areas, represent more humane substitutions for animals in these settings, and – with continued scientific effort – may change the conduct of basic research, clinical studies, safety testing, and drug development. Here, we present an introduction to these more human-relevant methodologies and suggest how a suite of pregnancy associated feto-maternal interface system-oriented NAMs may be integrated as reliable partial-/full animal replacements for investigators, significantly aid animal-/environmental welfare, and improve healthcare outcomes.

## Introduction

Since at least the fourth century Before Current Era, non-human animals have represented a large and important feature of biomedical research (1). While that history has occasionally been punctuated by the scientific victimization of certain human populations (condemned criminals, victims of war crimes, slaves, and orphans), revulsion by and rejection of such human experimentation by the majority of cultures left non-human animals as the default organisms for studying human physiology and pathophysiology (1, 2). During the more than one-thousand years before general anesthesia, there is no question that the conduct of such studies involved a ghastly experience for the unfortunate non-human. It is unlikely that early investigators accepted the Cartesian view of animals as automatons (3), and more likely that they steeled their reasoning (if not sentiment) to these agonized animal subjects as the best experimental substitute for their own species. Over many centuries, different groups advocated for changes to – even abandonment of – this practice known as vivisection. Arguably, the most important legislation for protecting animals used in laboratory settings originated in the 19th-century (4, 5) and has continued to this date (5). The recently ratified Food and Drug Administration (FDA) Modernization Act (6) contains an element of animal protection by strengthening consideration of validated non-animal methods in new drug regulatory submissions and by removing the requirement of animal testing for biosimilars. While no legislation has promoted either animal use or animal replacement, it is important to understand the advantages and limitations of each, if the best science is to be pursued.

## Animal models

The history of animal use in research extends over more than one and one-half millennia, while microphysiological systems (MPS) are relative infants. Clearly, there has been far more opportunity to scrutinize the value of animal use than that of MPS and our comments herein solely focus on the scientific value of each methodology. Animals are complete biological organisms, while MPS exist as single, or multiple (less than complete organism constituency) organ systems is created in specific formats by its designers. The representation of an entire, very complex biology is the largest advantage of animal use over presently available MPS (7, 8). Conversely, despite the phenotypic resemblance of many animal models to humans, mechanistic differences very often exist at the cellular level. Additionally, animal models differ in endocrine, paracrine, and immunological aspects to humans and there are structural differences of intrauterine organs compared to humans. Non-human primate models are the closest to human pregnancy; however, their use in pregnancy research is limited due to the high cost of conducting experiments to generate statistically and biologically relevant data, scarcity in obtaining the right models, and the duration of pregnancy. This leads to translatability problems, which are their largest disadvantage. In response, breeders of some animals used in laboratories will genetically manipulate their stock to more closely “humanize” them. However, as we learn more about the influence of other factors such as the microbiome and silent genetic mutations, this still may not be enough to “idealize” these animal models. The use of systematic reviews may have some role in mitigating this lack of similarity, by optimizing selection of well-defined non-human experimental subjects. Regardless of these negatives attached to animal models, they are the only complete near-human representations that we have currently. For that reason, they have maintained our attention and attract us to their use.

## Non-animal methods/new approach methods

A variety of MPS fill the categories of new approach methods (NAMs). MPS are engineered microenvironments that recapitulate the function of one or more organ systems. Among these physiological and pathophysiological miniaturizations, there are those which are subject to microfluidic flow and certain external forces and others in which those features are absent. A complete review and analysis of these types of systems (9) is beyond the scope of this article, and we will limit our discussion to the general groups of MPS and their most common applications. Our review is to introduce the concept and utility of NAMs and we restrict this review to describe NAMs developed to study feto-maternal interface (FMi) biology.

## Two-dimensional cell culture systems

The present majority of cell culture models are those having two dimensions (2D). They are the flat microscopic worlds familiar to most investigators. On the downside, they do not approximate the three-dimensional (3D) construct that is the human body, they soil and damage their own space, and their predictivity for drug development can be quite poor. Clearly, a methodological upgrade would be valuable and MPS developers have created and continue to create more human-relevant 3D systems.

## Three-dimensional organ systems

Spheroids represent the entry level for 3D organ systems. They are cultured as free-floating, spontaneously aggregating (single cell or multicellular mixtures adhering to each other), minimally complex and spherical cellular designs (10). They derive from immortalized cell lines, primary cells, or human tissue fragments and have found use in drug testing, nanoparticle examination, and the study of neurodegenerative diseases and liver physiology/pathophysiology (11). Heterogenous in nature, spheroids contain layers of proliferating and non-proliferating cells; of important note is the often-necrotic core of larger spheroids, which results from diffusion difficulties of nutrients and waste across their structures. However, this is a problem that also provides an opportunity to the study of drug delivery to the similarly necrotic cores of solid tumors (12, 13).

Another tiny workhorse is the organoid, a microengineered, self-assembling cluster of organ-specific cells arising from adult stem cells, embryonic stem cells, or induced pluripotent stem cells (iPSCs) (such as those from skin or blood which have been reprogrammed into an embryonic-like pluripotent state) (14). Unlike the cellular monocultures from which spheroids are derived, the variety of stem cells producing organoids can self-renew and differentiate into multiple lineages *in vitro*; this renewal process is responsible for an organoid’s greater longevity. While patient-derived tumor organoids and patient-derived spheroids (tumorspheres) have a place in cancer research and precision medicine strategies, the more functionally advanced organoid can be sourced as a living tissue biobank, provide disease-specific models, and (like some spheroids) be useful in drug screening (15–17). The spheroid and organoids are now being developed and getting used for several FMi research (18–27). These approaches and tools will be optimized and available widespread for research use in reproductive biology and medicine.

Organs-on-chips and Tissue-chips are the most sophisticated of the MPS (28, 29). In addition to having 3D tissue architecture and multiple cell type composition, they are subject to biomechanical forces and linked by microfluidics, which permit accurate recapitulation of certain key aspects of human physiology (30). This superiority to 2D models and the less advanced 3D models (especially, viewed in consideration of the reliability and translatability problems associated with animal use), should encourage development of this MPS category as a pathway toward developing species of interest (human) methodologies. The challenges and limitations of such development have been reviewed elsewhere (31) and we will only herein comment that they are not yet engineered to full human physiological complexity. Like animal models, MPS can have reproducibility problems associated with cellular and other system variability. These difficulties might diminish with improved standardization of cellular and technical components. Additionally helpful are resources such as that of the aggregated human and animal exposure data contained within the publicly available University of Pittsburgh Microphysiology Systems Database (MPS Db),^1^ which can aid in the selection of the best Organ-on-chip system for a particular study. Validation of Organs-on-chips, like other *in vitro* methods, is of current developer and regulatory interest, but a recent international roundtable of MPS stakeholders (privileged communication-Second BWF MPS Roundtable) noted that animal studies have not been subject to the same level of validation scrutiny as that proposed for MPS. Such thought prompts continued study of human MPS in various contexts, including its use in certain studies of reproductive physiology/pathophysiology.

## Pregnancy (maternal-fetal interface) physiology NAMs

Pregnancy is a unique condition where two independent physiological systems, the fetus and the mother, co-exist for a defined period to maintain pregnancy and aid fetal growth and development (32). This co-existence ends with parturition, a unique synchronized process that terminates all homeostatic states of pregnant uterine tissues (33–37). Unfortunately, an unacceptable and growing number of pregnancies do not end at term with the delivery of a fully developed fetus due to various adverse pregnancy outcomes (APOs). Currently, mechanistic knowledge of complex physiologic interactions between fetal and maternal tissue that maintain pregnancy and potential drug actions to treat APOs is unavailable. Due to this knowledge gap, therapeutic interventions are limited during pregnancy, making women and their fetuses a highly vulnerable population. The impact of maternal exposures like drugs or vaccines (efficacy and toxicology) during pregnancy is difficult to measure as longitudinal sampling of intrauterine tissues and other biological specimens for analysis is impractical. Similarly, various environmental exposures (pollutants and toxicants) disrupting pregnancy homeostasis and contributing to APOs are also difficult to predict. This lack of knowledge creates a major clinical dilemma during pregnancy, and the only option is to deliver the fetus prematurely, contributing to almost 1 million neonatal deaths around the globe annually (38–44).

Pregnancy complications and outcomes like preterm births are difficult to predict due to heterogeneities in the risk factors and biomolecular pathways that are effectors of preterm labor. One of the major risk factors of pregnancy complications are environmental pollutants and toxicants (45–56). An extensive study on pregnant subjects that tested a broad array of 59 chemicals reported that 80% of the chemicals were commonly found in both maternal and fetal samples (57), suggesting a maternal-fetal transport and potential harm to pregnancies (58). Unfortunately, epidemiological association between exposure and APOs have not reduced PTB rates stemming from such exposures. To better understand the impact of hazardous substances on pregnancy risk, a molecular mechanistic knowledge by which toxicants activate pathways causing preterm birth in maternal-fetal tissues are needed. Animal models have not been successful in determining pathological events contributing to adverse events during pregnancy, primarily due to the differences in the endocrine and paracrine systems between animal models and humans. Current *in vitro* toxicity testing methods cannot be used to accurately assess the hazard of tested substances on pregnancy outcomes as homeostatic disturbances resulting in immune imbalances at the feto-maternal interfaces cannot be measured using current approaches. Exposure to environmental toxicants often occurs in the form of mixed chemicals, especially during environmental disasters. However, current hazard assessment is conducted by evaluating single chemical at a time (59). It is well understood that simply combining the effect of individual chemicals cannot accurately determine the effect of mixed chemicals (60, 61), a major gap and challenge that is also recognized and prioritized (62, 63). It was found that, in some cases, when comparing toxic effects of a mixture of chemicals, the combined effects was greater than simply adding up the effects of the individual components (64, 65). These results strongly indicate that testing of actual environmental samples is desirable, rather than assuming that the effects of individual components from a mixed sample can be simply added together.

Therefore, reducing the risk of APOs is a global need (40, 66); however, pregnant women are excluded from most clinical trials and remain therapeutic orphans due to the lack of evidence for proper intervention (67–71). Challenges in conducting research and drug development, toxicology testing in pregnancy include (i) recruitment hurdles due to the paucity of data needed to convince the clinicians, subjects, and regulators of the utility of a drug in pregnancy, (ii) absence of informative biomarkers to assess the feto-placental response to therapeutics or other exposures, and (iii) lack of suitable preclinical models (*in vitro* or animal models) to address both pharmacokinetics and pharmacodynamics. These limitations lead to the systematic exclusion of pregnant women from clinical trials (68, 72).

Maintenance of human pregnancy and initiation of parturition requires complex coordination of cells among the fetal (placenta and fetal membranes)-maternal (cervix, decidua, and myometrium) organs (F-M). Therefore, a critical challenge to a reproductive scientist is modeling the coordinated inter-tissue and intercellular interactions among various F-M uterine systems, especially at the FMi tissues and their multiple functions that maintain a harmonious state of pregnancy. Current studies using 2D cell cultures (73–75), mixed co-cultures (76–78), transwell co-cultures (79–81), tissue explants (75, 82), organoids (83–85), organ baths (86), and *in vivo*, animal models (87, 88) have provided valuable information on respective organ systems’ contributions to pregnancy maintenance and some insights into malfunctions associated with adverse pregnancy events (89). However, limitations associated with these models are far more than reliable information needed for regulatory agencies to act on an intervention or a biomarker to reduce the incidences of APOs. The primary limitations of current cell culture models (2D or 3D and transwells) are: (1) these models do not provide intercellular interactions required to understand the homeostasis mechanisms during pregnancy. Transwells are still limited to two cell type models, (2) primary cells currently used do not survive many passages to replicate data, (3) difficulty to control inter individual variability of cells when tissues are collected from different subjects for cell isolation, and (many cell lines are not derived from pregnancies (e.g., choriocarcinoma cell lines used as the placental cell) and do not reflect true physiology expected during pregnancy, and (4) explant cultures can generate plethora of data; however, still cellular manipulations (gene knockout studies, cellular interaction studies, cell type specific response studies, etc.) are difficult.

Improving women’s reproductive health and fetal and neonatal outcome require physiologically relevant and experimentally manipulatable models that can generate high throughput data to study the human intrauterine organ system. Recent advances in our group’s efforts to create a suite of five pregnancy and women’s health-focused human MPS that model “healthy” and “disease” states of intrauterine tissues (90–97) present a unique opportunity to translate them into drug development tools. A biomimetic “organ-on a-chip (OOC)” recapitulates the multi-cellular organ system as seen *in vivo* using *in vitro* cell culture systems. These models can better mimic organ systems’ structure, functions, and responses, though they do not mean maintaining or growing actual organs on a chip. The purpose of OOC is not to build a whole living organ but to synthesize minimal functional units that recapitulate tissue- and organ-level functions (98). The combination of microfabrication, microfluidics, and iPSC technologies has provided many physiological models that better mimic human anatomy, functions, and responses more accurately, as seen *in vivo* (9, 99). These MPS can provide compartmentalized chambers that enable culturing and organizing cellular, extracellular matrices (ECMs), and other microenvironmental layers within these compartments while still propagating cellular signals, and sometimes even cells themselves, to propagate between the compartments through interconnected fluid paths. Animal models do not always mimic human pregnancy, particularly parturition (100, 101) and preterm birth induced in many of these models is not a naturally occurring pathobiological process (102). To overcome this, microphysiologic systems (OOC technology platform) has been used to recreate the placenta (103–108), fetal membrane (90, 96, 109–113), cervix (94, 114), vagina (115, 116), and blood–brain barrier models (117–120).

**FIGURE 1:**
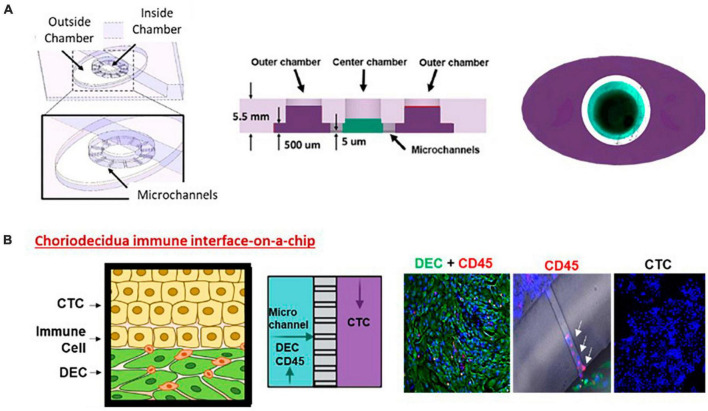
Choriodecidua interface (CD-OOC). **(A)** Three-dimensional and cross-sectional view of the two-chamber device. Cross-sectional view showing the diffusion barrier formation by liquid height difference. A dye-filled device with an outer chamber filled purple and an inner chamber filled green. **(B)** Adaptation of the two-chamber OOC to model the CDI. Cartoon showing the cell layers of the CDI along with a schematic depicting cell location within the OOC. Representative fluorescent images of vimentin + DEC cells (green) and CD45+ leukocytes in the inner chamber (red). Migratory leukocytes were identified by their CD45 staining (red) within microchannels (white arrows). Chorion cells in the outer chamber were only stained with DAPI (blue).

**FIGURE 2:**
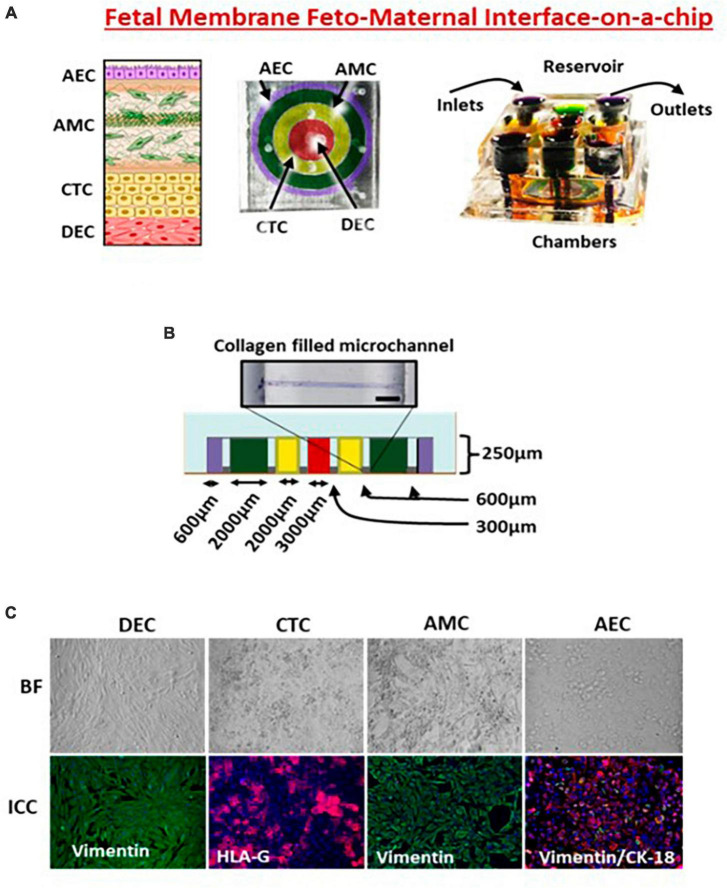
Feto-maternal interface (FMi-OOC). **(A)** FMi-OOC mimicking the fetal membrane amniochorion-decidua interface. FMi-OOC contains four circular chambers separated by arrays of microchannels. The cells are seeded as follows: decidua cells (red), CTCs (yellow), AMCs (green), and AECs (purple). Integrated media reservoir filled with color dye in each cell culture layer. **(B)** The width and height of chambers/microchannels. Collagen was stained with Masson trichome for visualization. **(C)** Brightfield and fluorescence images showing morphology, cytoskeletal markers (vimentin green; cytokeratin-18 red), and human leukocyte antigen G (HLA-G) in the chorion.

**FIGURE 3:**
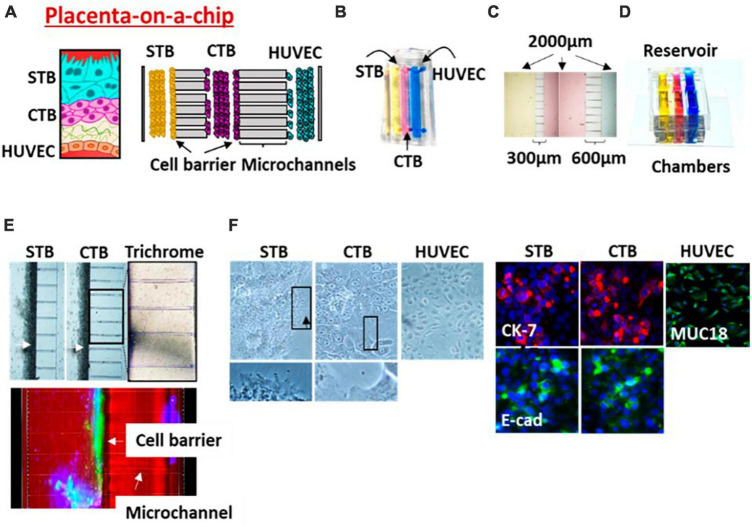
Placenta (PLA-OOC). **(A)** Schematic of the placenta trophoblast-endothelial interface. PLA-OOC contains three rectangular chambers separated by arrays of microchannels. **(B)** Color dye-injected PLA-OOC for easy visualization of each cell culture chamber. **(C)** Cell culture chambers showing the width and microchannel length. **(D)** Media reservoir layer that was aligned on top of the cell loading inlets and outlets. **(E)** Brightfield image showing STB and CTB cell barrier (white arrow) formation covering the microchannels. The cell barriers were confirmed to contain tight junction marker E-cadherin (green) expression confirming their functionality. Additionally, microchannels between the CTB and HUVEC chamber are filled with type I collagen to recreate the placenta stroma. Collagen was stained with Masson trichome for visualization (blue color, right image). **(F)** Brightfield and fluorescence images documents cell morphology, microvilli expression (black arrow), cytoskeletal markers [cytokeratin-7 (CK-7; red); tight junction marker (E-cadherin; green); and endothelial cell marker (MUC18; green)].

**FIGURE 4:**
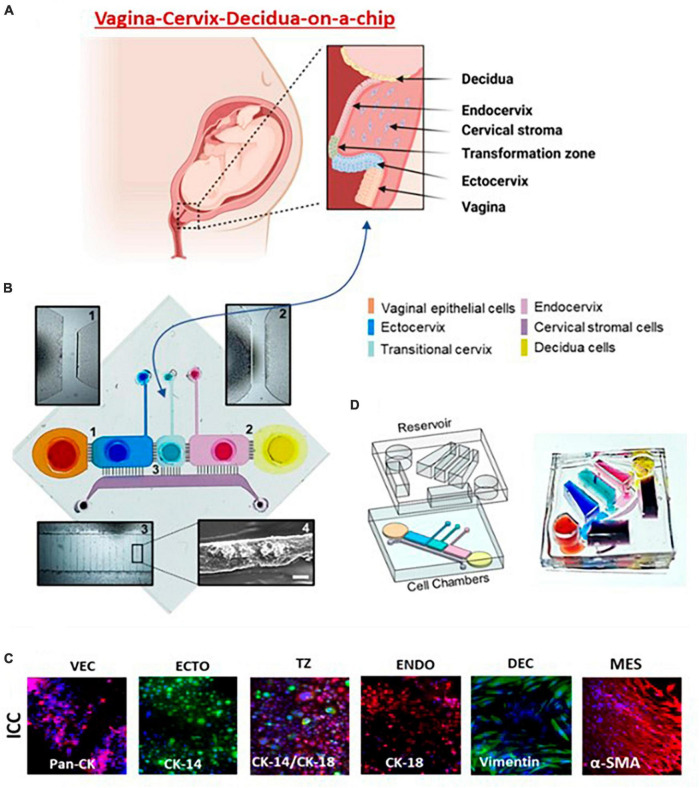
Vagina-cervix-decidua: VCD-OOC. **(A)** An illustration of the anatomy and histology of the female reproductive tract showing the vagina, cervix, and decidua. The epithelial cells of the vagina are continuous with the ectocervix, transformation zone, and endocervix. Beneath the epithelial layer is the cervical stromal layer. During term gestation, the fetal membrane, specifically the decidua, which is its outermost layer, lies directly above the endocervix. **(B)** Schematic illustration of the VCD-OOC showing the six cell culture chambers filled with color dye in each of the corresponding cell culture layers. (1) Brightfield microscopy of microchannels connecting the VEC-ECTO chambers; (2) brightfield microscopy image of microchannels connecting the ENDO-DEC chambers; (3) brightfield image showing the microchannels between the cervical epithelial cell chambers and the MES chamber filled with Matrigel; and (4) representative SEM image showing the type IV collagen in the microchannels. **(C)** Representative fluorescence microscopy images showing intermediate filaments and cell-specific markers [pan-cytokeratin (pan-CK), cytokeratin (CK)-14, CK-18, vimentin, α-smooth muscle actin (α-SMA), mucin (Muc5a), and type I collagen] of the cells cultured in VCD-OOC. **(D)** The VCD-OOC device with the integrated media reservoir filled with a dye in each cell culture layer for visualization.

Our MPS models of various intrauterine organ systems can address the limitations of existing models and ultimately accelerate preclinical drug development and provide reliable data for clinical trials. MPS models have been in use in other branches of medicine and are extensively recommended for preclinical trials (121, 122); however, reproductive medicine and the women’s health field have not yet advanced in utilizing MPS. Such MPS models can also accelerate the repurposing of already approved drugs during pregnancy. These futuristic approaches using the *non-animal model*s (also referred as NAMs) provide the next best opportunity for advancing research and filling knowledge gaps in the perinatal and reproductive health field. The basic design of each MPS model is that they have a (1) cell culture layer having cell culture chambers connected with arrays of microchannels and a (2) reservoir layer having large media reservoirs, where this layer is placed on top of the cell culture layer to support cell culture for 48–72 h without the need for media exchange. All the devices fit within a well of a 6-well plate (Figures 1–4; 92, 94–96, 112, 113) and can be operated with pipetting. The microfabrication of these MPS devices from polydimethylsiloxane (PDMS) has been extensively tested and validated (89, 90, 91, 112, 92, 94–96, 123–127).

## Currently available MPS – NAM and their main directions in the field of maternal-fetal physiology

### Modeling healthy and disease states using the feto-maternal interface on a chip

We extensively reported on the use of FMi-OOC (Figure 2) for modeling various maternal and fetal exposures and using a four-chamber device. During pregnancy, the fetal membrane (amniochorion) lines the intrauterine cavity and provides mechanical, immune, and endocrine support to the growing fetus (128). The fetal membranes also form one of the FMi by connecting to the maternal decidua. No MPS models have recreated this important FMi until now. The FMi-OOC chip has four concentric circles for cell cultures (96). Each chamber is 250 μm in height, and the width of each chamber was designed to mimic the thickness of each maternal and fetal layer [decidua, chorion, and amnion (mesenchyme and epithelium)] as seen *in utero.* This design allowed us to test four different cell types in four separate microenvironments (different culture mediums). Type IV collagen filled microchannels modeled the basement membranes and connected the chambers aligned with a media reservoir. This device contains maternal decidua (DEC) in the center chamber (green), connected to fetal chorion trophoblast cells (CTC) (yellow), amnion mesenchymal cells (AMC; pink), and amnion epithelial cells (AECs; purple) in the outer chamber. CTC and AMC layers are grown in a semi-3D suspension with Matrigel and primary amnion membrane-derived collagen to provide biological context (91, 92, 95, 96). This is the first MPS device to model the fetal membrane FMi allowing us to recreate disease phenotypes and assess the role of the *fetal inflammatory response*, a major determinant of adverse pregnancies and fetal morbidities. This device has been utilized to model ascending infection and inflammation [lipopolysaccharide (LPS) (96) and *Ureaplasma parvum*], identify the biological role of fetal-derived exosomes on labor induction (95), validate the functional role of drug transport proteins within fetal membrane cells (126), environmental toxicology studies (92), and conduct a preclinical trial with pravastatin and rosuvastatin (91). Results from these studies provide cellular and effluent biomarkers of various disease states and drug pharmacokinetics (PK), metabolism, and efficacy. Most importantly, the findings from FMi-OOC studies on infection-associated inflammatory changes leading to preterm birth and exosome-mediated feto-maternal communication, associated inflammatory changes, and parturition were replicated physiologically using animal models validating the usefulness of MPS devices as an alternative to animal models. To note, physiological validation in animal models has its own limitations, as they do not mimic human pregnancy and or parturition.

### Testing cigarette smoke and dioxin exposure induced cellular changes on feto-maternal cells

A two-chamber model of AEC and DEC separated by a semipermeable membrane was used to recreate FMi. Cells were treated with cigarette smoke extract (CSE) or dioxin for 48 h. The same experiments were conducted in transwells for comparison. Compared to transwell cultures, cells in OOC produced better membrane permeability regardless of the side of exposure (fetal vs. maternal). Membrane permeabilization was higher in AECs directly treated with CSE (1.6-fold) compared to similar treatment on the decidual side (1.2-fold). Both CSE and dioxin treatment on the maternal side induced cellular senescence on the fetal side. This effect was minimal in the transwell system. This confirmed the hypothesis that maternal oxidative stress (induced by cigarette smoke) can potentially cause premature aging of the fetal cells.

### Testing exposures by the mother or fetus and its pathologic impact on fetal cells

Limitations of the two-chamber model, where we cannot include all the cellular components of the FMi, promoted us to develop the four-chamber device. FMi-OOC was used extensively by our lab to report the impact of cigarette smoke and Cadmium toxicity.

### Cigarette smoke causes sterile inflammation, as seen in non-infectious inflammatory conditions in preterm birth

Using FMi-OOC, we recreated an environment induced by cigarette smoke extract (CSE – oxidative stress inducer). Maternal and fetal CSE exposures induced the following: (1) time-dependent propagation of cigarette smoke as measured by nicotine in the media in various cell chambers, (2) cellular transitions [epithelial mesenchymal transition (EMT)] similar to that seen in pathologic preterm birth, (3) senescence of cells induced by oxidative stress, and (4) sterile inflammation characterized by increased proinflammatory cytokines and reduced antiinflammatory cytokines. These changes were similar to our prior reports using the animal models (129), suggesting that MPS models are ideal for replacing animal models in experimental reproductive biology.

### Cadmium toxicity and fetal outcome

Exposure to environmental chemicals can prematurely trigger labor-initiating signals at the FMi, leading to spontaneous preterm birth. Using Cadmium as a model toxicant, we tested the effect of maternal exposure to Cadmium (Cd) (92). Cd transport through the FMi and its impact on the cell cycle, cell death, and inflammation were analyzed. Cd treatment resulted in significant cell death and a pro-inflammatory environment in the maternal decidua but had minimal effect on the fetal chorion cells and no effect in the fetal amnion cells compared to controls. The mother primarily mediated the inflammatory response and not the fetus. The maternal response, but lack of fetal response, indicates that Cd-mediated adverse effects originate from maternal pathophysiology rather than fetal-derived triggers of preterm labor. This study demonstrates that the FMi-OOC can indeed predict the response of FMi upon exposure to chemicals, opening the possibility for using OOC models for environmental toxin screens (92). Compared with cigarette smoke exposure described above, these data suggest that FMi-OOC can determine the differential effects, i.e., the pathological triggers (pathways and biomarkers) that contribute to APOs are maternal or fetal driven. This knowledge is critical in determining intervention strategies.

### Extracellular vesicle (exosomes) mediated communication in response to toxicant exposure

Maternal and fetal exposure to various pollutants and toxicants can lead to premature senescence of the feto-maternal FMi tissues (130–133). These pathological changes prematurely produce exosomes that cargo cellular damage associated inflammatory mediators. These exosomes are communication channels between the mother and the fetus and often cause functional changes, including labor (131, 133). We tested the feto-maternal communication using FMi-OOC where exosomes carrying High mobility group box 1 protein [HMGB1; one of the damages associated molecular pattern marker (DAMP)] related changes were tested. Exosomes with HMGB1 is hypothesized to increase the inflammatory load at the FMi (95). To test this, exosomes from AECs grown under normal conditions were engineered to contain HMGB1 by electroporation (eHMGB1). eHMGB1 propagation through FMi was tested using FMi-OOC. We have reported that eHMGB1 propagated through the fetal cells to the maternal decidua and increased inflammation [receptor expression (RAGE and TLR4) and cytokines]. Furthermore, intra-amniotic injection of eHMGB1 (containing 10 ng) into pregnant CD-1 mice on embryonic day 17 led to PTB. Injecting carboxyfluorescein succinimidyl ester (CFSE)-labeled eHMGB1, we determined *in vivo* kinetics. We reported that eHMGB1 trafficking resulting in preterm birth in animal models was also associated with increased FMi inflammation, such as that we observed in cells of the FMi-OOC. This study provided *in vivo* functional validation of FMi-OOC experiments and strengthened the reliability of such devices to test physiologic and pathologic systems (95). Bioengineered microvesicles (particles with higher size than exosomes) are also ideal for studying biochemical gradients and compartmentalized responses in *in vitro* models. Besides the FMi MPS models described above, other microphysiologic platforms exist, such as the placenta on a chip. This two-chamber device that contained two cells types (one from choriocarcinoma and other HUVEC cells also contained ECM) (105). Variations of these designs have been used by other investigators (104, 134–138). Some of these models are used for environmental toxicology trials.

The MPS based on NAMs have notable strengths. MPS models that can recreate an entire organ can overcome the limitations of many of the current approaches (2D cultures, explant models, transwell models, organoids, and animal models). MPS models require lesser number of cells and media compared to traditional approaches. Cellular migration, propagation of paracrine factors (e.g., extracellular vesicles) can be easily studies using MPS platforms. Creation of a healthy and disease states of an organ can mimic the state of the organ *in vivo* and can be used to determine pathophysiology at different cellular levels in an organ, discover biomarkers of the pathophysiology, and test efficacy of compounds that can reinstate the healthy state of the organ.

With all of this said, these MPS are not without limitations. Although the architecture of the organ can be reproduced in an *in vitro* model, they are difficult to manufacture, and miniaturized cell culture chambers are difficult to operate. PDMS used for many of the currently used MPS models have been reported to absorb certain compounds and this may pose a limitation to study specific secreted products or incorporating these substances in experimental models. Translational research requires high throughput and large volume of data generation and most academic research lab settings may depend on commercial sources for their design and manufacture, prior to conducting any experiment; this can be cost prohibitive. Low number of cells and media used may also pose difficulties in terms of having sufficient materials to conduct several routine lab experiments (e.g., Western blot analysis). However, many labs have now designed ways to overcome many of these limitations and certain technological advancements have helped to reduce the sample volume required to conduct several lab tasks.

## A future for NAMs

Reliability, reproducibility, and translatability are the features needed to make NAMs part of the research mainstream. As we have written before, this will require them to perform at a level that satisfies the needs of investigators (139). Reproducing the features and function of complex biological systems is an inescapably daunting task. If the past work of developers is any indicator for future advances in NAMs, then we should anticipate an equally awesome evolutionary process. Along that creative timeline, input from other stakeholders is crucial. While regulators will remain as the gatekeepers for releasing end products, conversations among the stakeholder groups most closely associated with the challenges confronting NAMs will have relevance for informing and driving the developmental process. The initial core demographics of academic scientists, industry, government funders, government regulators, venture capital, philanthropy, animal research advocacy, and animal protection, should be joined by patient advocacy groups, specialty clinicians, ethicists, and other stakeholders holding significant interests in the promise that NAMs have for delivering improved healthcare outcomes, increased scientific accuracy, reduced and/or eliminated reliance on animal use, and optimized environmental welfare. NAMs are ideal but still there are limitations to accessing them and conducting experiments using them. For example, microfluidic and other platforms are not accessible to several labs and trained personnel to conduct experiments are also currently lacking. Human primary cells, cell lines or human iPSCs are also needed for use in these experimental models. Accessibility or cost associated in accessing them may also limit several researchers from acquiring these technologies or using them.

## Author contributions

RM: Conceptualization, Data curation, Funding acquisition, Writing – original draft, Writing – review and editing. LL: Conceptualization, Supervision, Writing – original draft, Writing – review and editing. LM: Conceptualization, Writing – original draft, Writing – review and editing.

## Footnotes

1
https://upddi.pitt.edu/microphysiology-systems-database/

## References

[1] Donnelley S Nolan K. Animals, science, and ethics – pre face; introduction: the troubled middle in medias res; future directions. *Hastings Cent Rep.* (1990) 20:S1–4. 11650361

[2] Ross MW. The ethics of experiments on higher animals. *Soc Sci Med.* (1981) 15F:51–60. 11655144

[3] Guerrini A. The ethics of animal experimentation in seventeenth-century England. *J Hist Ideas*. (1989) 50:391–407. 11645864

[4] Balls M. Scientific procedures on living animals: proposals for reform of the 1876 Cruelty to Animals Act. *Altern Lab Anim*. (1985) 12:225–42. 12085921

[5] Lancet. Animals in research. *Lancet.* (1985) 1:1230. 11644485

[6] Han JJ. FDA Modernization Act 2.0 allows for alternatives to animal testing. *Artif Organs*. (2023) 47:449–50. 10.1111/aor.14503 36762462

[7] Malik M Yang Y Fathi P Mahler GJ Esch MB. Critical Considerations for the Design of Multi-Organ Microphysiological Systems (MPS). *Front Cell Dev Biol*. (2021) 9:721338. 10.3389/fcell.2021.721338 34568333 PMC8459628

[8] Khazali AS Clark AM Wells AA. Pathway to Personalizing Therapy for Metastases Using Liver-on-a-Chip Platforms. *Stem Cell Rev Rep*. (2017) 13:364–80. 10.1007/s12015-017-9735-3 28425064 PMC5484059

[9] Ingber DE. Human organs-on-chips for disease modelling, drug development and personalized medicine. *Nat Rev Genet*. (2022) 23:467–91. 10.1038/s41576-022-00466-9 35338360 PMC8951665

[10] Hargrove-Grimes P Low LA Tagle DA. Microphysiological systems: stakeholder challenges to adoption in drug development. *Cells Tissues Organs*. (2022) 211:269–81. 10.1159/000517422 34380142 PMC8831652

[11] Białkowska K Komorowski P Bryszewska M Miłowska K. Spheroids as a type of three-dimensional cell cultures-examples of methods of preparation and the most important application. *Int J Mol Sci*. (2020) 21:6225. 10.3390/ijms21176225 32872135 PMC7503223

[12] Ryu NE Lee SH Park H. Spheroid culture system methods and applications for mesenchymal stem cells. *Cells.* (2019) 8:1620. 10.3390/cells8121620 31842346 PMC6953111

[13] Millard M Yakavets I Zorin V Kulmukhamedova A Marchal S Bezdetnaya L. Drug delivery to solid tumors: the predictive value of the multicellular tumor spheroid model for nanomedicine screening. *Int J Nanomedicine*. (2017) 12:7993–8007. 10.2147/IJN.S146927 29184400 PMC5673046

[14] Amiel-Pérez J Amiel-Sáenz J Amiel-Torrelio M. Organoids: fundamentals, present and future. *Rev Peru Med Exp Salud Publica*. (2022) 39:227–35. 10.17843/rpmesp.2022.392.10203 36477325 PMC11397781

[15] Rossi M Alviano F Righi S Sabattini E Agostinelli C. Three-dimensional models: a novel approach for lymphoma research. *J Cancer Res Clin Oncol*. (2022) 148:753–65. 10.1007/s00432-021-03897-9 35091834 PMC11801009

[16] Bassi G Grimaudo MA Panseri S Montesi M. Advanced multi-dimensional cellular models as emerging reality to reproduce in vitro the human body complexity. *Int J Mol Sci*. (2021) 22:1195. 10.3390/ijms22031195 33530487 PMC7865724

[17] Rodrigues J Heinrich MA Teixeira LM Prakash J. 3D in vitro model (R)evolution: unveiling tumor-stroma interactions. *Trends Cancer*. (2021) 7:249–64. 10.1016/j.trecan.2020.10.009 33218948

[18] Zhuang BM Cao DD Li TX Liu XF Lyu MM Wang SD Single-cell characterization of self-renewing primary trophoblast organoids as modeling of EVT differentiation and interactions with decidual natural killer cells. *BMC Genomics*. (2023) 24:618. 10.1186/s12864-023-09690-x 37853336 PMC10583354

[19] Karvas RM Theunissen TW. Generation of 3D trophoblast organoids from human naïve pluripotent stem cells. *Methods Mol Biol*. (2023) 496. 10.1007/7651_2023_496 37402094 PMC10766861

[20] Yang L Semmes EC Ovies C Megli C Permar S Gilner JB Innate immune signaling in trophoblast and decidua organoids defines differential antiviral defenses at the maternal-fetal interface. *eLife*. (2022) 11:e79794. 10.7554/eLife.79794 35975985 PMC9470165

[21] Yang L Liang P Yang H Coyne CB. Trophoblast organoids with physiological polarity model placental structure and function. *J Cell Sci*. (2024) 137:jcs261528. 10.1242/jcs.261528 37676312 PMC10499031

[22] Paris F Marrazzo P Pizzuti V Marchionni C Rossi M Michelotti M Characterization of perinatal stem cell spheroids for the development of cell therapy strategy. *Bioengineering (Basel).* (2023) 10:189. 10.3390/bioengineering10020189 36829683 PMC9952228

[23] Stojanovska V Arnold S Bauer M Voss H Fest S Zenclussen AC. Characterization of three-dimensional trophoblast spheroids: an alternative model to study the physiological properties of the placental unit. *Cells*. (2022) 11:2884. 10.3390/cells11182884 36139458 PMC9497053

[24] Qiu Q Li Y Fong SW Lee KC Chen ACH Ruan H Endometrial stromal cells from women with repeated implantation failure display impaired invasion towards trophoblastic spheroids. *Reproduction*. (2023) 165:335–46. 10.1530/REP-22-0282 36656637

[25] Alexandrova M Manchorova D You Y Mor G Dimitrova V Dimova T. Functional HLA-C expressing trophoblast spheroids as a model to study placental-maternal immune interactions during human implantation. *Sci Rep.* (2022) 12:10224. 10.1038/s41598-022-12870-6 35715452 PMC9205925

[26] Huang W Fong SW Yeung WSB Lee YL. Human Trophectoderm Spheroid Derived from Human Embryonic Stem Cells. *Methods Mol Biol*. (2022) 2520:181–7. 10.1007/7651_2021_460 35218527

[27] Zhou W Barton S Cui J Santos LL Yang G Stern C Infertile human endometrial organoid apical protein secretions are dysregulated and impair trophoblast progenitor cell adhesion. *Front Endocrinol (Lausanne).* (2022) 13:1067648. 10.3389/fendo.2022.1067648 36589798 PMC9794621

[28] Rogal J Schlünder K Loskill P. Developer’s guide to an organ-on-chip model. *ACS Biomater Sci Eng*. (2022) 8:4643–7. 10.1021/acsbiomaterials.1c01536 35760397 PMC9667877

[29] Ko J Park D Lee S Gumuscu B Jeon NL. Engineering organ-on-a-chip to accelerate translational research. *Micromachines (Basel).* (2022) 13:1200. 10.3390/mi13081200 36014122 PMC9412404

[30] Low LA Mummery C Berridge BR Austin CP Tagle DA. Organs-on-chips: into the next decade. *Nat Rev Drug Discov*. (2021) 20:345–61. 10.1038/s41573-020-0079-3 32913334

[31] Vunjak-Novakovic G Ronaldson-Bouchard K Radisic M. Organs-on-a-chip models for biological research. *Cell*. (2021) 184:4597–611. 10.1016/j.cell.2021.08.005 34478657 PMC8417425

[32] Romero R Dey SK Fisher SJ. Preterm labor: one syndrome, many causes. *Science*. (2014) 345:760–5. 10.1126/science.1251816 25124429 PMC4191866

[33] Tal R Taylor HS. Endocrinology of pregnancy. In: Feingold KR Anawalt B Boyce A editors. *Endotext.* South Dartmouth, MA: MDText.com, Inc (2000).

[34] Rokas A Mesiano S Tamam O LaBella A Zhang G Muglia L. Developing a theoretical evolutionary framework to solve the mystery of parturition initiation. *eLife*. (2020) 9:e58343. 10.7554/eLife.58343 33380346 PMC7775106

[35] Smith R. Alterations in the hypothalamic pituitary adrenal axis during pregnancy and the placental clock that determines the length of parturition. *J Reprod Immunol*. (1998) 39:215–20. 10.1016/s0165-0378(98)00023-0 9786463

[36] Menon R Bonney EA Condon J Mesiano S Taylor RN. Novel concepts on pregnancy clocks and alarms: redundancy and synergy in human parturition. *Hum Reprod Update*. (2016) 22:535–60. 10.1093/humupd/dmw022 27363410 PMC5001499

[37] Mendelson CR Montalbano AP Gao L. Fetal-to-maternal signaling in the timing of birth. *J Steroid Biochem Mol Biol*. (2017) 170:19–27. 10.1016/j.jsbmb.2016.09.006 27629593 PMC5346347

[38] Beck S Wojdyla D Say L Betran AP Merialdi M Requejo JH The worldwide incidence of preterm birth: a systematic review of maternal mortality and morbidity. *Bull World Health Organ*. (2010) 88:31–8. 10.2471/BLT.08.062554 20428351 PMC2802437

[39] Shapiro-Mendoza CK Lackritz EM. Epidemiology of late and moderate preterm birth. *Semin Fetal Neonatal Med*. (2012) 17:120–5. 10.1016/j.siny.2012.01.007 22264582 PMC4544710

[40] Simmons LE Rubens CE Darmstadt GL Gravett MG. Preventing preterm birth and neonatal mortality: exploring the epidemiology, causes, and interventions. *Semin Perinatol*. (2010) 34:408–15. 10.1053/j.semperi.2010.09.005 21094415

[41] Blencowe H Cousens S Oestergaard MZ Chou D Moller AB Narwal R National, regional, and worldwide estimates of preterm birth rates in the year 2010 with time trends since 1990 for selected countries: a systematic analysis and implications. *Lancet*. (2012) 379:2162–72. 10.1016/S0140-6736(12)60820-4 22682464

[42] Dammann O Leviton A. Maternal intrauterine infection, cytokines, and brain damage in the preterm newborn. *Pediatr Res*. (1997) 42:1–8. 10.1203/00006450-199707000-00001 9212029

[43] Jacobsson B. Infectious and inflammatory mechanisms in preterm birth and cerebral palsy. *Eur J Obstet Gynecol Reprod Biol*. (2004) 115:159–60. 10.1016/j.ejogrb.2003.11.014 15262348

[44] Muglia LJ Katz M. The enigma of spontaneous preterm birth. *N Engl J Med*. (2010) 362:529–35. 10.1056/NEJMra0904308 20147718

[45] Manzano-León N Quintana R Sánchez B Serrano J Vega E Vázquez-López I Variation in the composition and in vitro proinflammatory effect of urban particulate matter from different sites. *J Biochem Mol Toxicol.* (2013) 27:87–97. 10.1002/jbt.21471 23335408 PMC4355014

[46] Vadillo-Ortega F Osornio-Vargas A Buxton MA Sánchez BN Rojas-Bracho L Viveros-Alcaráz M Air pollution, inflammation and preterm birth: a potential mechanistic link. *Med Hypotheses.* (2014) 82:219–24. 10.1016/j.mehy.2013.11.042 24382337 PMC3928635

[47] Ferguson KK McElrath TF Meeker JD. Environmental phthalate exposure and preterm birth. *JAMA Pediatr.* (2014) 168:61–7. 10.1001/jamapediatrics.2013.3699 24247736 PMC4005250

[48] Jalaludin B Mannes T Morgan G Lincoln D Sheppeard V Corbett S. Impact of ambient air pollution on gestational age is modified by season in Sydney, Australia. *Environ Health*. (2007) 6:16. 10.1186/1476-069X-6-16 17553174 PMC1894960

[49] Ritz B Wilhelm M Hoggatt KJ Ghosh JK. Ambient air pollution and preterm birth in the environment and pregnancy outcomes study at the University of California, Los Angeles. *Am J Epidemiol.* (2007) 166:1045–52. 10.1093/aje/kwm181 17675655

[50] Hohmann C Grabenhenrich L de Kluizenaar Y Tischer C Heinrich J Chen CM Health effects of chronic noise exposure in pregnancy and childhood: a systematic review initiated by ENRIECO. *Int J Hyg Environ Health.* (2013) 216:217–29. 10.1016/j.ijheh.2012.06.001 22854276

[51] Schifano P Lallo A Asta F De Sario M Davoli M Michelozzi P. Effect of ambient temperature and air pollutants on the risk of preterm birth, Rome 2001-2010. *Environ Int.* (2013) 61:77–87. 10.1016/j.envint.2013.09.005 24103349

[52] Wilhelm M Ritz B. Local variations in CO and particulate air pollution and adverse birth outcomes in Los Angeles County, California, USA. *Environ Health Perspect.* (2005) 113:1212–21. 10.1289/ehp.7751 16140630 PMC1280404

[53] Zhao Q Liang Z Tao S Zhu J Du Y. Effects of air pollution on neonatal prematurity in Guangzhou of China: a time-series study. *Environ Health*. (2011) 10:2. 10.1186/1476-069X-10-2 21214958 PMC3024279

[54] Salihu HM Ghaji N Mbah AK Alio AP August EM Boubakari I. Particulate pollutants and racial/ethnic disparity in feto-infant morbidity outcomes. *Matern Child Health J.* (2012) 16:1679–87. 10.1007/s10995-011-0868-8 21833758

[55] Le HQ Batterman SA Wirth JJ Wahl RL Hoggatt KJ Sadeghnejad A Air pollutant exposure and preterm and term small-for-gestational-age births in Detroit, Michigan: long-term trends and associations. *Environ Int.* (2012) 44:7–17. 10.1016/j.envint.2012.01.003 22314199 PMC4331339

[56] Trasande L Wong K Roy A Savitz DA Thurston G. Exploring prenatal outdoor air pollution, birth outcomes and neonatal health care utilization in a nationally representative sample. *J Expo Sci Environ Epidemiol.* (2013) 23:315–21. 10.1038/jes.2012.124 23340702 PMC4391972

[57] Morello-Frosch R Cushing LJ Jesdale BM Schwartz JM Guo W Guo T Environmental chemicals in an urban population of pregnant women and their newborns from San Francisco. *Environ Sci Technol.* (2016) 50:12464–72. 10.1021/acs.est.6b03492 27700069 PMC6681912

[58] Lam J Sutton P Kalkbrenner A Windham G Halladay A Koustas E A systematic review and meta-analysis of multiple airborne pollutants and autism spectrum disorder. *PLoS One*. (2016) 11:e0161851. 10.1371/journal.pone.0161851 27653281 PMC5031428

[59] Clahsen SCS van Kamp I Hakkert BC Vermeire TG Piersma AH Lebret E. Why do countries regulate environmental health risks differently? A theoretical perspective. *Risk Anal.* (2019) 39:439–61. 10.1111/risa.13165 30110518 PMC7379724

[60] Kortenkamp A Faust M. Regulate to reduce chemical mixture risk. *Science*. (2018) 361:224–6. 10.1126/science.aat9219 30026211

[61] Zhang YH Liu SS Liu HL Liu ZZ. Evaluation of the combined toxicity of 15 pesticides by uniform design. *Pest Manag Sci.* (2010) 66:879–87. 10.1002/ps.1957 20602526

[62] Carlin DJ Rider CV Woychik R Birnbaum LS. Unraveling the health effects of environmental mixtures: an NIEHS priority. *Environ Health Perspect.* (2013) 121:A6–8. 10.1289/ehp.1206182 23409283 PMC3553446

[63] Rider CV Carlin DJ Devito MJ Thompson CL Walker NJ. Mixtures research at NIEHS: an evolving program. *Toxicology*. (2013) 313:94–102. 10.1016/j.tox.2012.10.017 23146757 PMC4232209

[64] Chen Z Lloyd D Zhou YH Chiu WA Wright FA Rusyn I. Risk characterization of environmental samples using in vitro bioactivity and polycyclic aromatic hydrocarbon concentrations data. *Toxicol Sci.* (2021) 179:108–20. 10.1093/toxsci/kfaa166 33165562 PMC7797768

[65] Hsieh NH Chen Z Rusyn I Chiu WA. Risk characterization and probabilistic concentration-response modeling of complex environmental mixtures using new approach methodologies (NAMs) data from organotypic in vitro human stem cell assays. *Environ Health Perspect*. (2021) 129:17004. 10.1289/EHP7600 33395322 PMC7781439

[66] McCabe ER Carrino GE Russell RB Howse JL. Fighting for the next generation: US Prematurity in 2030. *Pediatrics*. (2014) 134:1193–9. 10.1542/peds.2014-2541 25367536

[67] McKiever M Frey H Costantine MM. Challenges in conducting clinical research studies in pregnant women. *J Pharmacokinet Pharmacodyn*. (2020) 47:287–93. 10.1007/s10928-020-09687-z 32306165 PMC8237366

[68] Illamola SM Bucci-Rechtweg C Costantine MM Tsilou E Sherwin CM Zajicek A. Inclusion of pregnant and breastfeeding women in research - efforts and initiatives. *Br J Clin Pharmacol*. (2018) 84:215–22. 10.1111/bcp.13438 28925019 PMC5777434

[69] Zimmerman K Gonzalez D Swamy GK Cohen-Wolkowiez M. Pharmacologic studies in vulnerable populations: using the pediatric experience. *Semin Perinatol*. (2015) 39:532–6. 10.1053/j.semperi.2015.08.007 26358805 PMC4628576

[70] Committee Opinion Acog. No. 646: ethical considerations for including women as research participants. *Obstet Gynecol*. (2015) 126:e100–7. 10.1097/AOG.0000000000001150 26488521

[71] Sheffield JS Siegel D Mirochnick M Heine RP Nguyen C Bergman KL Designing drug trials: considerations for pregnant women. *Clin Infect Dis.* (2014) 59(Suppl 7):S437–44. 10.1093/cid/ciu709 25425722 PMC4303056

[72] Gonzalez D Boggess KA Cohen-Wolkowiez M. Lessons learned in pediatric clinical research to evaluate safe and effective use of drugs in pregnancy. *Obstet Gynecol*. (2015) 125:953–8. 10.1097/AOG.0000000000000743 25751205 PMC4372491

[73] Lavu N Richardson L Radnaa E Kechichian T Urrabaz-Garza R Sheller-Miller S Oxidative stress-induced downregulation of glycogen synthase kinase 3 beta in fetal membranes promotes cellular senescence†. *Biol Reprod*. (2019) 101:1018–30. 10.1093/biolre/ioz119 31292604 PMC7150613

[74] Moore JJ Dubyak GR Moore RM Vander Kooy D. Oxytocin activates the inositol-phospholipid-protein kinase-C system and stimulates prostaglandin production in human amnion cells. *Endocrinology*. (1988) 123:1771–7. 10.1210/endo-123-4-1771 3138102

[75] Myatt L Rosenfield RB Eis AL Brockman DE Greer I Lyall F. Nitrotyrosine residues in placenta. Evidence of peroxynitrite formation and action. *Hypertension*. (1996) 28:488–93. 10.1161/01.hyp.28.3.488 8794838

[76] Hochberg A Sibley C Pixley M Sadovsky Y Strauss B Boime I. Choriocarcinoma cells increase the number of differentiating human cytotrophoblasts through an in vitro interaction. *J Biol Chem*. (1991) 266:8517–22. 2022665

[77] Presicce P Senthamaraikannan P Alvarez M Rueda CM Cappelletti M Miller LA Neutrophil recruitment and activation in decidua with intra-amniotic IL-1beta in the preterm rhesus macaque. *Biol Reprod*. (2015) 92:56. 10.1095/biolreprod.114.124420 25537373 PMC4342792

[78] Yang F Zheng Q Jin L. Dynamic function and composition changes of immune cells during normal and pathological pregnancy at the maternal-fetal interface. *Front Immunol*. (2019) 10:2317. 10.3389/fimmu.2019.02317 31681264 PMC6813251

[79] Kumar D Moore RM Mercer BM Mansour JM Mesiano S Schatz F In an in-vitro model using human fetal membranes, 17-α hydroxyprogesterone caproate is not an optimal progestogen for inhibition of fetal membrane weakening. *Am J Obstet Gynecol.* (2017) 217:.e1–695. 10.1016/j.ajog.2017.10.004 29031893

[80] Wong MK Li EW Adam M Selvaganapathy PR Raha S. Establishment of an in vitro placental barrier model cultured under physiologically relevant oxygen levels. *Mol Hum Reprod*. (2020) 26:353–65. 10.1093/molehr/gaaa018 32159799 PMC7227181

[81] Zaga-Clavellina V Garcia-Lopez G Flores-Herrera H Espejel-Nuñez A Flores-Pliego A Soriano-Becerril D In vitro secretion profiles of interleukin (IL)-1beta, IL-6, IL-8, IL-10, and TNF alpha after selective infection with *Escherichia coli* in human fetal membranes. *Reprod Biol Endocrinol*. (2007) 5:46. 10.1186/1477-7827-5-46 18078521 PMC2175507

[82] Fortunato SJ Menon R Swan KF Lyden TW. Organ culture of amniochorionic membrane in vitro. *Am J Reprod Immunol*. (1994) 32:184–7. 10.1111/j.1600-0897.1994.tb01112.x 7880402

[83] Alzamil L Nikolakopoulou K Turco MY. Organoid systems to study the human female reproductive tract and pregnancy. *Cell Death Differ*. (2021) 28:35–51. 10.1038/s41418-020-0565-5 32494027 PMC7852529

[84] Cui Y Zhao H Wu S Li X. Human female reproductive system organoids: applications in developmental biology, disease modelling, and drug discovery. *Stem Cell Rev Rep*. (2020) 16:1173–84. 10.1007/s12015-020-10039-0 32929605

[85] Wei Y Zhang C Fan G Meng L. Organoids as novel models for embryo implantation study. *Reprod Sci*. (2021) 28:1637–43. 10.1007/s43032-021-00501-w 33650092

[86] Mörlin B Andersson E Byström B Hammarström M. Nitric oxide induces endometrial secretion at implantation time. *Acta Obstet Gynecol Scand*. (2005) 84:1029–34. 10.1111/j.0001-6349.2005.00804.x 16232168

[87] Gokina NI Fairchild RI Bishop NM Dawson TE Prakash K Bonney EA. Kinetics of postpartum mesenteric artery structure and function relative to pregnancy and lactation in mice. *Reprod Sci*. (2021) 28:1200–15. 10.1007/s43032-020-00402-4 33415648 PMC7935827

[88] Spencer NR Radnaa E Baljinnyam T Kechichian T Tantengco OAG Bonney E Development of a mouse model of ascending infection and preterm birth. *PLoS One*. (2021) 16:e0260370. 10.1371/journal.pone.0260370 34855804 PMC8638907

[89] Radnaa E Richardson L Goldman B Burks JK Baljinnyam T Vora N Stress signaler p38 mitogen-activated kinase activation: a cause for concern? *Clin Sci (Lond).* (2022) 136:1591–614. 10.1042/CS20220491 36250628 PMC9664350

[90] Richardson L Kim S Menon R Han A. Organ-on-chip technology: the future of feto-*maternal* interface research? *Front Physiol*. (2020) 11:715. 10.3389/fphys.2020.00715 32695021 PMC7338764

[91] Richardson LS Kammala A Costantine MM Fortunato SJ Radnaa E Kim S. Testing of drugs using human feto-maternal interface organ-on-chips provide insights into pharmacokinetics and efficacy. *Lab Chip*. (2022) 22:4574–92. 10.1039/d2lc00691j 36322152 PMC9682442

[92] Kim S Richardson L Radnaa E Chen Z Rusyn I Menon R Molecular mechanisms of environmental toxin cadmium at the feto-maternal interface investigated using an organ-on-chip (FMi-OOC) model. *J Hazard Mater*. (2022) 422:126759. 10.1016/j.jhazmat.2021.126759 34391970 PMC8595660

[93] Tantengco OAG Richardson LS Vink J Kechichian T Medina PMB Pyles RB Progesterone alters human cervical epithelial and stromal cell transition and migration: Implications in cervical remodeling during pregnancy and parturition. *Mol Cell Endocrinol*. (2021) 529:111276. 10.1016/j.mce.2021.111276 33823217 PMC8491272

[94] Tantengco OAG Richardson LS Medina PMB Han A Menon R. Organ-on-chip of the cervical epithelial layer: a platform to study normal and pathological cellular remodeling of the cervix. *FASEB J*. (2021) 35:e21463. 10.1096/fj.202002590RRR 33689188 PMC8193817

[95] Radnaa E Richardson LS Sheller-Miller S Baljinnyam T de Castro Silva M Kumar Kammala A Extracellular vesicle mediated feto-maternal HMGB1 signaling induces preterm birth. *Lab Chip*. (2021) 21:1956–73. 10.1039/d0lc01323d 34008619 PMC8162392

[96] Richardson LS Kim S Han A Menon R. Modeling ascending infection with a feto-maternal interface organ-on-chip. *Lab Chip*. (2020) 20:4486–501. 10.1039/d0lc00875c 33112317 PMC7815379

[97] Richardson L Vargas G Brown T Ochoa L Trivedi J Kacerovský M Redefining 3Dimensional placental membrane microarchitecture using multiphoton microscopy and optical clearing. *Placenta*. (2017) 53:66–75. 10.1016/j.placenta.2017.03.017 28487023

[98] Bhatia SN Ingber DE. Microfluidic organs-on-chips. *Nat Biotechnol*. (2014) 32:760–72. 10.1038/nbt.2989 25093883

[99] Bai H Ingber DE. What can anorgan-on-a-chip teach us about human lung pathophysiology? *Physiology (Bethesda).* (2022) 37. 10.1152/physiol.00012.2022 35658627 PMC9394778

[100] Manuel CR Ashby CR Reznik SE. Discrepancies in animal models of preterm birth. *Curr Pharm Des*. (2017) 23:6142–8. 10.2174/1381612823666171012101114 29022513

[101] Nielsen BW Bonney EA Pearce BD Donahue LR Sarkar IN. A cross-species analysis of animal models for the investigation of preterm birth mechanisms. *Reprod Sci*. (2016) 23:482–91. 10.1177/1933719115604729 26377998 PMC5933186

[102] Nold C Maubert M Anton L Yellon S Elovitz MA. Prevention of preterm birth by progestational agents: what are the molecular mechanisms? *Am J Obstet Gynecol.* (2013) 208:.e1–7. 10.1016/j.ajog.2013.01.020 23433326 PMC3581865

[103] Blundell C Yi YS Ma L Tess ER Farrell MJ Georgescu A Placental drug transport-on-a-chip: a microengineered in vitro model of transporter-mediated drug efflux in the human placental barrier. *Adv Healthc Mater.* (2018) 7:10.1002/adhm.201700786. 10.1002/adhm.201700786 29121458 PMC5793852

[104] Horii M Touma O Bui T Parast MM. Modeling human trophoblast, the placental epithelium at the maternal fetal interface. *Reproduction*. (2020) 160:R1–11. 10.1530/REP-19-0428 32485667 PMC7286067

[105] Lee JS Romero R Han YM Kim HC Kim CJ Hong JS Placenta-on-a-chip: a novel platform to study the biology of the human placenta. *J Matern Fetal Neonatal Med*. (2016) 29:1046–54. 10.3109/14767058.2015.1038518 26075842 PMC5625348

[106] Pemathilaka RL Reynolds DE Hashemi NN. Drug transport across the human placenta: review of placenta-on-a-chip and previous approaches. *Interface Focus*. (2019) 9:20190031. 10.1098/rsfs.2019.0031 31485316 PMC6710654

[107] Pemathilaka RL Caplin JD Aykar SS Montazami R Hashemi NN. Placenta-on-a-chip: in vitro study of caffeine transport across placental barrier using liquid chromatography mass spectrometry. *Glob Chall*. (2019) 3:1800112. 10.1002/gch2.201800112 31565368 PMC6436596

[108] Yin F Zhu Y Zhang M Yu H Chen W Qin J. A 3D human placenta-on-a-chip model to probe nanoparticle exposure at the placental barrier. *Toxicol In Vitro*. (2019) 54:105–13. 10.1016/j.tiv.2018.08.014 30248392

[109] Aronoff DM. Deconstructing extraplacental membranes to understand bacterial chorioamnionitis. *Trans Am Clin Climatol Assoc.* (2020) 131:72–9. 32675845 PMC7358510

[110] Gnecco JS Anders AP Cliffel D Pensabene V Rogers LM Osteen K Instrumenting a fetal membrane on a chip as emerging technology for preterm birth research. *Curr Pharm Des*. (2017) 23:6115–24. 10.2174/1381612823666170825142649 28847303

[111] Dixon CL Richardson L Sheller-Miller S Saade G Menon R. A distinct mechanism of senescence activation in amnion epithelial cells by infection, inflammation, and oxidative stress. *Am J Reprod Immunol.* (2018) 79:10.1111/aji.12790. 10.1111/aji.12790 29193446 PMC5815890

[112] Richardson L Jeong S Kim S Han A Menon R. Amnion membrane organ-on-chip: an innovative approach to study cellular interactions. *FASEB J.* (2019) 33:8945–60. 10.1096/fj.201900020RR 31039044 PMC6662977

[113] Richardson L Gnecco J Ding T Osteen K Rogers LM Aronoff DM Fetal membrane organ-on-chip: an innovative approach to study cellular interactions. *Reprod Sci.* (2019) 21:1933719119828084. 10.1177/1933719119828084 30791822

[114] Khorsandi D Palacios S Gaslain Y Emsellem C Combalia J Cortés J P159 Human uterine cervix-on-a-chip: establishing the first in vitro model to study the development of cervical carcinoma and human papiloma virus mechanism of action. *Int J Gynecol Cancer.* (2019) 29.

[115] Mancini V Pensabene V. Organs-On-Chip Models *of* the Female *Reproductive* System. *Bioengineering (Basel).* (2019) 6:103. 10.3390/bioengineering6040103 31703369 PMC6956296

[116] Xiao S Coppeta JR Rogers HB Isenberg BC Zhu J Olalekan SA A microfluidic culture model of the human reproductive tract and 28-day menstrual cycle. *Nat Commun*. (2017) 8:14584. 10.1038/ncomms14584 28350383 PMC5379057

[117] Jagadeesan S Workman MJ Herland A Svendsen CN Vatine GD. Generation of a human iPSC-based blood-brain barrier chip. *J Vis Exp.* (2020): 10.3791/60925 32176199

[118] Jeong S Kim S Buonocore J Park J Welsh CJ Li J A three-dimensional arrayed microfluidic blood-brain barrier model with integrated electrical sensor array. *IEEE Trans Biomed Eng*. (2018) 65:431–9. 10.1109/TBME.2017.2773463 29346110 PMC11233983

[119] van der Helm MW van der Meer AD Eijkel JC van den Berg A Segerink LI. Microfluidic organ-on-chip *technology* for blood-brain barrier research. *Tissue Barriers*. (2016) 4:e1142493. 10.1080/21688370.2016.1142493 27141422 PMC4836466

[120] Vatine GD Barrile R Workman MJ Sances S Barriga BK Rahnama M Human iPSC-derived blood-brain barrier chips enable disease modeling and personalized medicine applications. *Cell Stem Cell.* (2019) 24:995.e–1005.e. 10.1016/j.stem.2019.05.011 31173718

[121] Prantil-Baun R Novak R Das D Somayaji MR Przekwas A Ingber DE. Physiologically based pharmacokinetic and pharmacodynamic analysis enabled by microfluidically linked organs-on-chips. *Annu Rev Pharmacol Toxicol*. (2018) 58:37–64. 10.1146/annurev-pharmtox-010716-104748 29309256

[122] Benam KH Dauth S Hassell B Herland A Jain A Jang KJ Engineered in vitro disease models. *Annu Rev Pathol*. (2015) 10:195–262. 10.1146/annurev-pathol-012414-040418 25621660

[123] Richardson LS Emezienna N Burd I Taylor BD Peltier MR Han A Adapting an organ-on-chip device to study the effect of fetal sex and maternal race/ethnicity on preterm birth related intraamniotic inflammation leading to fetal neuroinflammation. *Am J Reprod Immunol*. (2022) 88:e13638. 10.1111/aji.13638 36308737 PMC9712252

[124] Tantengco OAG Richardson LS Radnaa E Kammala AK Kim S Medina PMB Modeling ascending Ureaplasma parvum infection through the female reproductive tract using vagina-cervix-decidua-organ-on-a-chip and feto-maternal interface-organ-on-a-chip. *FASEB J*. (2022) 36:e22551. 10.1096/fj.202200872R 36106554 PMC9500016

[125] Tantengco OAG Richardson LS Radnaa E Kammala AK Kim S Medina PMB Exosomes from Ureaplasma parvum-infected ectocervical epithelial cells promote feto-maternal interface inflammation but are insufficient to cause preterm delivery. *Front Cell Dev Biol*. (2022) 10:931609. 10.3389/fcell.2022.931609 36046342 PMC9420848

[126] Ganguly E Kammala AK Benson M Richardson LS Han A Menon R. Organic anion transporting polypeptide 2B1 in human fetal membranes: a novel gatekeeper for drug transport During pregnancy? *Front Pharmacol*. (2021) 12:771818. 10.3389/fphar.2021.771818 34987396 PMC8721670

[127] Sheller-Miller S Radnaa E Yoo JK Kim E Choi K Kim Y Exosomal delivery of NF-κB inhibitor delays LPS-induced preterm birth and modulates fetal immune cell profile in mouse models. *Sci Adv.* (2021) 7:eabd3865. 10.1126/sciadv.abd3865 33523942 PMC10671068

[128] Benson-Martin J Zammaretti P Bilic G Schweizer T Portmann-Lanz B Burkhardt T *The* Young’s modulus of fetal preterm and term amniotic membranes. *Eur J Obstet Gynecol Reprod Biol*. (2006) 128:103–7. 10.1016/j.ejogrb.2005.12.011 16442204

[129] Polettini J Richardson LS Menon R. Oxidative stress induces senescence and sterile inflammation in murine amniotic cavity. *Placenta*. (2018) 63:26–31. 10.1016/j.placenta.2018.01.009 29486853 PMC5833301

[130] Sheller-Miller S Radnaa E Arita Y Getahun D Jones RJ Peltier MR Environmental pollutant induced cellular injury is reflected in exosomes from placental explants. *Placenta*. (2020) 89:42–9. 10.1016/j.placenta.2019.10.008 31675489 PMC7024050

[131] Shahin HI Radnaa E Tantengco OAG Kechichian T Kammala AK Sheller-Miller S Microvesicles and exosomes released by amnion epithelial cells under oxidative stress cause inflammatory changes in uterine cells†. *Biol Reprod*. (2021) 105:464–80. 10.1093/biolre/ioab088 33962471 PMC8335356

[132] Sheller-Miller S Urrabaz-Garza R Saade G Menon R. Damage-Associated molecular pattern markers HMGB1 and cell-Free fetal telomere fragments in oxidative-Stressed amnion epithelial cell-Derived exosomes. *J Reprod Immunol*. (2017) 123:3–11. 10.1016/j.jri.2017.08.003 28858636 PMC5632595

[133] Shepherd MC Radnaa E Tantengco OA Kechichian T Urrabaz-Garza R Kammala AK Extracellular vesicles from maternal uterine cells exposed to risk factors cause fetal inflammatory response. *Cell Commun Signal*. (2021) 19:100. 10.1186/s12964-021-00782-3 34620169 PMC8499538

[134] James JL Lissaman A Nursalim YNS Chamley LW. Modelling human placental villous development: designing cultures that reflect anatomy. *Cell Mol Life Sci*. (2022) 79:384. 10.1007/s00018-022-04407-x 35753002 PMC9234034

[135] Abostait A Tyrrell J Abdelkarim M Shojaei S Tse WH El-Sherbiny IM Placental nanoparticle uptake-*on*-a-chip: the impact of trophoblast syncytialization and shear stress. *Mol Pharm*. (2022) 19:3757–69. 10.1021/acs.molpharmaceut.2c00216 36053057

[136] Rabussier G Bünter I Bouwhuis J Soragni C van Zijp T Ng CP Healthy and diseased placental barrier on-a-chip models suitable for standardized studies. *Acta Biomater*. (2023) 164:363–76. 10.1016/j.actbio.2023.04.033 37116636

[137] Luconi M Sogorb MA Markert UR Benfenati E May T Wolbank S Human-based new approach methodologies in developmental toxicity testing: a step ahead from the state of the art with a feto-placental organ-on-chip platform. *Int J Environ Res Public Health*. (2022) 19:15828. 10.3390/ijerph192315828 36497907 PMC9737555

[138] Costa J Mackay R de Aguiar Greca SC Corti A Silva E Karteris E The role of the 3Rs for understanding and modeling the human placenta. *J Clin Med*. (2021) 10:3444. 10.3390/jcm10153444 34362227 PMC8347836

[139] Levin LH Muglia LJ. Alternative thinking about animals in research. *NAM Perspect.* (2022) 2022:2022. 10.31478/202211a 36713772 PMC9875849

